# Supplementary material for: Loss of surface transport is a main cellular pathomechanism of CRB2 variants causing podocytopathies
Source: Life Sci Alliance. 2022 Dec 22;6(3):e202201649. doi: 10.26508/lsa.202201649 (PMC9780758; doi:10.26508/lsa.202201649)
Supplement: Supplementary file 2 [file LSA-2022-01649_TableS2.docx]

**Suppl. Table S2:** *Quantification of cells with CRB2 at the PM.* Percentage of cells in the cell population, which show CRB2-GFP signal at the PM. If a cell showed any GFP signal at the PM, it was grouped to “PM” (N=3, n>300 cells).

| **CRB2 variant** | **cells with CRB2 signal at the PM[%]** | **cells without CRB2 signal at the PM [%]** |
| --- | --- | --- |
| *reference and wildtypes (benign) variants* | | |
| WT | 86.76 | 13.24 |
| M145T | 84.98 | 15.02 |
| R610W | 85.47 | 14.53 |
| *deletion mutant* | | |
| ΔEGF10 | 8.08 | 91.92 |
| *disease-associated CRB2 variants* | | |
| C384F | 4.51 | 95.49 |
| R534W | 5.72 | 94.28 |
| C620S | 4.93 | 95.07 |
| R628C | 5.68 | 94.32 |
| C629S | 8.9 | 91.1 |
| R633W | 12.53 | 87.47 |
| E643A | 5.99 | 94.01 |
| N800K | 9.79 | 90.21 |
| P1064S | 13.53 | 86.47 |
| R1072C | 32.21 | 67.79 |
| G1088D | 4.29 | 95.71 |
| T1187P | 3.88 | 96.12 |
| G1205S | 6.26 | 93.74 |
| R1249Q | 20.74 | 79.26 |
| *additional cysteine variants:* | | |
| C614S | 8.58 | 91.42 |
| C631R | 3.64 | 96.36 |
| C614Y | 6.89 | 93.11 |
| C631F | 6.17 | 93.83 |
