# Supplementary material for: Loss of surface transport is a main cellular pathomechanism of CRB2 variants causing podocytopathies
Source: Life Sci Alliance. 2022 Dec 22;6(3):e202201649. doi: 10.26508/lsa.202201649 (PMC9780758; doi:10.26508/lsa.202201649)
Supplement: Supplementary file 3 [file LSA-2022-01649_TableS3.docx]

**Suppl. Table S3: *Putative disulfide bridges in the CRB2 region containing LG1, LG2 and EGF10***

Disulfide bonds formed in LG1 (579↔603), EGF10 (609↔620, 614↔629 and 631↔640) and LG2 (766↔805) in cysteine affected variants of CRB2 EGF10. Calculations are based on *RosettaFold* protein modeling. All variants form the same disulfide bonds in the LG domains. In contrast to wt, mutations affecting a cysteine residue lead to a missing disulfide bond (only two bonds in EGF10) or formation of another combination of the second bond in case of C614S/Y and C629S. Variants with additional cysteine residues in EGF10 do not alter the number of disulfide bonds. (+ = predicted bond at this position, - = no bond predicted). CRB2 disulfide positions base on Uniprot sequence Q5IJ48.

|  |  | CRB2 variants | | | | | | | | | |
| --- | --- | --- | --- | --- | --- | --- | --- | --- | --- | --- | --- |
| disulfide bonds | domain | WT | R605C | C614S | C614Y | C620S | F627C | R628C | C629S | C631F | C631R |
| predicted positions |  | | | | | | | | | | |
| C579 ↔ C603 | LG1 | + | + | + | + | + | + | + | + | + | + |
| C609 ↔ C620 | EGF10 | + | + | + | + | - | + | + | + | + | + |
| C614 ↔ C629 | EGF10 | + | + | - | - | + | + | + | - | + | + |
| C631 ↔ C640 | EGF10 | + | + | - | - | + | + | + | - | - | - |
| C766 ↔ C805 | LG2 | + | + | + | + | + | + | + | + | + | + |
| *de novo* positions |  | | | | | | | | | | |
| C629 ↔ C640 | EGF10 | - | - | + | + | - | - | - | - | - | - |
| C614 ↔ C640 | EGF10 | - | - | - | - | - | - | - | + | - | - |
