# Supplementary material for: Loss of surface transport is a main cellular pathomechanism of CRB2 variants causing podocytopathies
Source: Life Sci Alliance. 2022 Dec 22;6(3):e202201649. doi: 10.26508/lsa.202201649 (PMC9780758; doi:10.26508/lsa.202201649)
Supplement: Supplementary file 4 [file LSA-2022-01649_TableS4.docx]

**Suppl. Table S4:** *Primers for cloning and mutagenesis.*

| **Cloning** |  |  |  |
| --- | --- | --- | --- |
| **Target** | **Usage** | **Direction** | **Sequence (5’-3’)** |
| hCRB2_WT | amplification | forward | caccgggcgcgccatggcgctggccaggcctgggac |
| hCRB2_WT | amplification | reverse | ttaattaactagatgagtctctcctccggtgg |
| hCRB2_M145T | mutagenesis | forward | gtgacctgcgag**acg**gaggtggacg |
| hCRB2_M145T | mutagenesis | reverse | cgtccacctccgtc**tcg**caggtcac |
| hCRB2_C384F | mutagenesis | forward | gctatatctgcagg**ttc**ccagagacctgg |
| hCRB2_C384F | mutagenesis | reverse | ccaggtctctgg**gaa**cctgcagatatagc |
| hCRB2_R534W | mutagenesis | forward | ccctggagcta**tgg**ctctggcatgagg |
| hCRB2_R534W | mutagenesis | reverse | cctcatgccagag**cca**tagctccaggg |
| hCRB2_R610W | mutagenesis | forward | agagcagtgc**tgg**cctctgccttgtg |
| hCRB2_R610W | mutagenesis | reverse | cacaaggcagagg**cca**gcactgctct |
| hCRB2_C614S | mutagenesis | forward | ccggcctctgcct**tct**gtccacggaggg |
| hCRB2_C614S | mutagenesis | reverse | ccctccgtggac**aga**aggcagaggccgg |
| hCRB2_C614Y | mutagenesis | forward | ccggcctctgcct**tat**gtccacggaggg |
| hCRB2_C614Y | mutagenesis | reverse | ccctccgtggac**ata**aggcagaggccgg |
| hCRB2_C620S | mutagenesis | forward | ccacggagggtcc**tct**gtggatctgtgg |
| hCRB2_C620S | mutagenesis | reverse | ccacagatccac**aga**ggaccctccgtgg |
| hCRB2_R628C | mutagenesis | forward | ggactcatttc**tgt**tgcgactgtgccc |
| hCRB2_C628C | mutagenesis | reverse | gggcacagtcgca**aca**gaaatgagtcc |
| hCRB2_C629S | mutagenesis | forward | actcatttccgt**tct**gactgtgcc |
| hCRB2_C629S | mutagenesis | reverse | ggcacagtc**aga**acggaaatgagt |
| hCRB2_C631F | mutagenesis | forward | cgttgcgac**ttt**gcccggcccc |
| hCRB2_C631F | mutagenesis | reverse | ggggccgggc**aaa**gtcgcaacg |
| hCRB2_C631R | mutagenesis | forward | cgttgcgac**cgt**gcccggcccc |
| hCRB2_C631R | mutagenesis | reverse | ggggccgggc**acg**gtcgcaacg |
| hCRB2_R633W | mutagenesis | forward | tgcgactgtgcc**tgg**ccccatagagg |
| hCRB2_R633W | mutagenesis | reverse | cctctatgggg**cca**ggcacagtcgca |
| hCRB2_E643A | mutagenesis | forward | cacgtgcgctgat**gcg**attcctgctgccac |
| hCRB2_E643A | mutagenesis | reverse | gtggcagcaggaat**cgc**atcagcgcacgtg |
| hCRB2_N800K | mutagenesis | forward | ggcggcaggcagtcctgg**aag**ctcactgcgggctgcgtctcc |
| hCRB2_N800K | mutagenesis | reverse | ggagacgcagcccgcagtgag**ctt**ccaggactgcctgccgcc |
| hCRB2_P1064S | mutagenesis | forward | gtgcgccctcg**tcc**tgtctgcacgac |
| hCRB2_P1064S | mutagenesis | reverse | gtcgtgcagaca**gga**cgagggcgcac |
| hCRB2_R1072C | mutagenesis | forward | cggtgcctgc**tgt**gacctcttcgac |
| hCRB2_R1072C | mutagenesis | reverse | gtcgaagaggtc**aca**gcaggcaccg |
| hCRB2_G1088D | mutagenesis | forward | ggggtgggaa**gac**ccgcgctgcgaag |
| hCRB2_G1088D | mutagenesis | reverse | cttcgcagcgcgg**gtc**ttcccacccc |
| hCRB2_T1187P | mutagenesis | forward | gaatgggggc**ccc**tgccgggcag |
| hCRB2_T1187P | mutagenesis | reverse | ctgcccggca**ggg**gcccccattc |
| hCRB2_G1205S | mutagenesis | forward | gccagattctcc**agc**cagttctgtgaagtg |
| hCRB2_G1205S | mutagenesis | reverse | cacttcacagaactg**gct**ggagaatctggc |
| hCRB2_R1249Q | mutagenesis | forward | atcctggcagcc**caa**aagcgccg |
| hCRB2_R1249Q | mutagenesis | reverse | cggcgctt**ttg**ggctgccaggat |
| **Sequencing** |  |  |  |
| hCRB2_Seq_02F | sequencing | forward | ccttggctatgcaggcgtga |
| hCRB2_Seq_03F | sequencing | forward | tgtgcgaggtggacgaggacg |
| hCRB2_Seq_03R | sequencing | reverse | cgatgcacactcgtcctcgtc |
| hCRB2_Seq_05F | sequencing | forward | aggtttcgcaccacactgcc |
| hCRB2_Seq_06F | sequencing | forward | tgtggatggccacctcctgc |
| hCRB2_Seq_08F | sequencing | forward | tccgaggacatgtgcagtcc |
| hCRB2_Seq_10F | sequencing | forward | gaacttcaccggctgcttgg |
| hCRB2_Seq_11F | sequencing | forward | cgatcacatggtcctgctgg |
| hU6_TLCV2_F | sequencing, KO Plasmid, SantaCruz | reverse | gagggcctatttcccatgatt |
| hPDIA3_Intron_F | amplification, sequencing | forward | GAGTGGTGCAAGGCATTCTAAGG |
| hPDIA3_Exon7_R | amplification, sequencing | reverse | AAGCAATAAGTAAGTCCTTGCCCTG |
